# Multi-Objective Optimization of Low-Carbon Concrete Mix Proportions Considering Carbon Emissions and Creep Behavior

**DOI:** 10.3390/ma19214570

**Published:** 2026-10-29

**Authors:** Yunlong Wu, Houmin Li, Jinyan Xie

**Affiliations:** 1 School of Civil Engineering, Architecture and the Environment, Hubei University of Technology, Wuhan 430068, China; 2 Innovation Demonstration Base of Ecological Environment Geotechnical and Ecological Restoration of Rivers and Lakes, Hubei University of Technology, Wuhan 430068, China

**Keywords:** low-carbon concrete, creep prediction, physical prior, sparse variational Gaussian process, NSGA-II, SHAP

## Abstract

This study proposes a physical-prior and residual-learning method for creep prediction of low-carbon concrete and integrates it into a multi-objective optimization framework for mix proportion design. After cleaning and normalizing 1066 creep test records, the B4 model served as the physical prior backbone, and a sparse variational Gaussian process (SVGP) was used to fit the residuals between measured and theoretical values. Global linear calibration, a Matérn-5/2 kernel with automatic relevance determination, and length-scale regularization were used to suppress kernel collapse and overfitting. The B4+SVGP model was compared with random forest, extreme gradient boosting, and a backpropagation neural network under a unified dataset, feature set, and training protocol, and Shapley additive explanations (SHAP) were used to analyze mixture effects on creep compliance. The B4+SVGP model that performed best under record-level validation was then embedded in NSGA-II to optimize net carbon emissions and 1000-day creep compliance simultaneously; TOPSIS was used to derive mix proportions under carbon-emission-oriented, balanced, and creep-oriented weight preferences. Under a unified record-level 10-fold cross-validation protocol, B4+SVGP achieved favorable prediction accuracy (R^2^ = 0.9660) and retained a relative advantage in the target mix proportion region. Loading duration, age at loading, and 28-day compressive strength were the most influential factors, consistent with the physical mechanisms of the B4 model. Under strength and mix-feasibility constraints, the Pareto front exhibited an L shape with an inflection at approximately 205 kg/m^3^ net carbon emissions. The balanced (5:5) scenario yielded a candidate with a water-to-binder ratio of 0.4227, a binder content of 300.03 kg/m^3^, and fly ash/slag/silica fume replacements of 6.76%/6.70%/14.82%; it reduced net carbon emissions by approximately 18.7% while increasing creep compliance by only approximately 2.1%. The resulting mixtures are computational candidates whose fresh-state properties, compressive strength, and long-term creep behavior require experimental verification before engineering recommendation.

## 1. Introduction

Cementitious materials are a major source of emissions in global building decarbonization: concrete production accounts for approximately 7–9% of global anthropogenic CO_2_ emissions, with cement clinker production as the main contributor [1], and each tonne of ordinary Portland cement (OPC) releases approximately 0.8–0.9 tonnes of CO_2_ [2]. Partially replacing cement with fly ash, ground granulated blast-furnace slag (GGBFS), and other supplementary cementitious materials (SCMs) can reduce carbon emissions by 20–60% and improve workability [3,4]. However, SCMs also alter the creep behavior of concrete, thereby affecting long-term structural performance and safety [5,6].

Creep plays a decisive role in the long-term deflection, prestress loss, and internal force redistribution of long-span bridges, high-rise buildings, and other structures [7,8]. Although increasing the SCM content reduces carbon emissions, it may alter creep behavior and create a trade-off between carbon reduction and long-term deformation [9,10]. Consequently, understanding the creep behavior of low-carbon concrete and incorporating it into mix proportion design has become an urgent requirement for engineering applications.

The main approaches to low-carbon concrete include replacing cement with fly ash, slag, and silica fume, developing limestone calcined clay cement (LC3), and using recycled aggregates [1,11]. Adu-Amankwah [2] reported that ternary blended cement can reduce embodied carbon emissions from 0.178 to below 0.1 kg CO_2_e/kg, but a high clinker replacement ratio results in slow early strength development. Nukah et al. [12] reported a 365-day creep coefficient of only 0.019. This result indicates that creep can be substantially lower than conventional expectations under specific low-carbon mix proportions and curing conditions; however, its applicability remains constrained by mix proportion, age, and test conditions and should not be extrapolated directly to all low-carbon concrete systems. Robalo et al. [13] reduced the cement content to 175 kg/m^3^ while maintaining good durability, and creep was controlled by the equivalent water-to-cement ratio. Alghazali and Myers [14] found that ecological self-consolidating concrete with a 75% replacement ratio exhibited lower creep and shrinkage. The creep behavior of low-carbon concrete is highly sensitive to mix proportions, and the conclusions of existing studies remain inconsistent; thus, a unified understanding has not yet been established. In addition, studies on GGBFS-based geopolymer concrete and eco-friendly concrete incorporating natural zeolite indicate that mix proportion optimization and environmental assessment of low-carbon cementitious systems should consider both mechanical performance and service-environment impacts [15,16].

Mainstream empirical creep models (CEB-FIP, ACI 209, and B4) are mostly calibrated using OPC data, and their applicability to concrete with high SCM contents remains questionable [7,17]. Existing improvements include the following: Liu et al. [9] introduced K_L to quantify the effect of fly ash; Liang et al. [5] found through Bayesian inverse analysis that design codes underestimated the early creep of GGBFS concrete within 200 h; Chen et al. [18] developed strength and creep models for concrete with high SCM contents; and Šmilauer et al. [10] verified that the creep of blended cement containing 20% clinker remained within one standard deviation of the B4 and EC2 predictions. Bouras and Li [6] used machine learning to predict creep and confirmed that loading duration was the most significant variable. However, purely data-driven models require large datasets and lack physical constraints, making it difficult to strike a balance between small-sample performance and physical consistency.

From a materials-mechanism perspective, concrete creep is generally regarded as the combined result of viscous flow of the solid skeleton, pore-water migration, microcrack development, and microprestress relaxation [19,20,21]. Its evolution is simultaneously governed by age at loading, loading duration, environmental humidity, and microstructure. Therefore, creep is not only related to macroscopic strength but is also strongly coupled with loading history and internal moisture diffusion. A purely empirical regression model cannot fully characterize this multiscale, multifactor coupling behavior.

The integration of physical priors and machine learning can alleviate data scarcity. The physics-informed neural network (PINN) proposed by Raissi et al. [22] embeds governing equations in the loss function to achieve high-accuracy modeling with limited data. Wang et al. [23] used a physical model to provide baseline predictions and then captured higher-order features through residual learning. Gaussian process regression (GPR) combines small-sample modeling capability with uncertainty diagnostics through predictive variance [24], providing a basis for subsequent assessments of model reliability. However, studies on creep prediction that use the B4 model as a physical prior and correct its systematic bias through residual learning remain limited [25].

Multi-objective optimization has become an important tool for sustainable concrete design, and NSGA-II is the most widely used algorithm [26]. Existing frameworks mainly focus on strength, cost, and carbon emissions [27,28,29] but rarely consider creep. Although Kandiri and Fotouhi [30] included the creep compliance as an optimization objective, their optimization targeted model parameters rather than mix proportions.

To address these limitations, this study proposes a creep prediction method based on a physical prior and residual learning. The B4 model is used as the physical prior baseline, and Gaussian process regression is employed to fit the residuals between B4 predictions and measured values, thereby compensating for the systematic bias of the empirical model under limited data. A B4-prior-guided, SVGP-residual-corrected creep prediction model is constructed and embedded in NSGA-II as a surrogate model to obtain the Pareto-optimal set of low-carbon concrete mix proportions by simultaneously optimizing net carbon emissions per unit volume and 1000-day creep compliance. TOPSIS is then used to provide decision support for candidate mix proportions under different weight preferences.

## 2. Machine Learning and Multi-Objective Optimization Algorithms

### 2.1. Dataset and Data Preprocessing

The creep test data used in this study were obtained from the creep and shrinkage laboratory database developed by Vít Šmilauer and the concrete creep and shrinkage database of Northwestern University (NU) [31]. These databases aggregate standard creep test data measured by multiple laboratories worldwide over several decades and have good representativeness and coverage.

To ensure data quality, the original dataset was screened through four steps: field completeness, physical validity, unit consistency, and duplicate removal. First, records missing any required input feature or creep output were removed. Second, physically reasonable ranges were set for continuous inputs: the water-to-binder ratio, aggregate-to-binder ratio, cement content, SCM replacement ratios, age at loading, drying age, loading duration, relative humidity, ambient temperature, volume-to-surface-area ratio, and 28-day compressive strength were required to be positive or within engineering-reasonable ranges; records with non-positive creep strain were also excluded. Third, units were standardized, and records with obvious inconsistencies in mixture composition or time variables were removed. Finally, duplicate records were removed. Here, duplicates mainly include three types: identical entries within the same database; repeated records of the same specimen under identical loading, environmental, and measurement conditions; and identifiable duplicate reports of the same original test from different source databases or publications. For these duplicate records, the entry with the most complete fields was retained. Measurements of the same specimen at different loading durations were treated as distinct time-point samples and were not removed as duplicates. Cross-source records that could not be reliably identified as the same original test were not forcibly merged. Model residuals or target-value distributions were not used for outlier removal, thereby avoiding data leakage. After these steps, 1066 samples were retained. Table 1 summarizes the descriptive statistics of the main input features, including minimum, maximum, mean, and standard deviation. Except for studies cited in the literature review, the creep prediction target in this study is creep compliance J(t,t0), that is, the total strain response under unit stress; the term creep coefficient is used only to describe previous studies and is not used as the modeling or optimization output.

### 2.2. Data Normalization

Table 1 shows that the input parameters have different dimensions and value ranges. For models such as BPNN and SVGP that rely on gradient optimization or kernel distances, feature-scale differences affect numerical stability and distance metrics [32]; for tree-based models such as RF and XGBoost, feature scale does not affect split thresholds, and normalization is not an inherent prerequisite. To ensure consistent preprocessing across all models under the same dataset, feature set, and training protocol, all continuous input features were uniformly normalized using min-max scaling to the interval [0, 1]. The main purpose was to reduce the influence of preprocessing differences on model comparison. The normalization equation is:

(1) $$\begin{array}{c} {\tilde{X}}_{i}=\frac{X_{i}-X_{i,\min}}{X_{i,\max}-X_{i,\min}} \end{array}$$

where *x_i_* is the original value of the ith input feature; *x_i_*_,min_ and *x_i_*_,max_ are the minimum and maximum values of that feature in the training folds, respectively, and both are calculated only from training-fold data; and *x_i_*,norm is the normalized value. The output target was uniformly defined as creep compliance J(t,t0) and normalized in the same manner to match the model assumptions, and predictions were inverse-transformed to the original scale.

### 2.3. Selection and Encoding of Input Features

**Figure 1:**
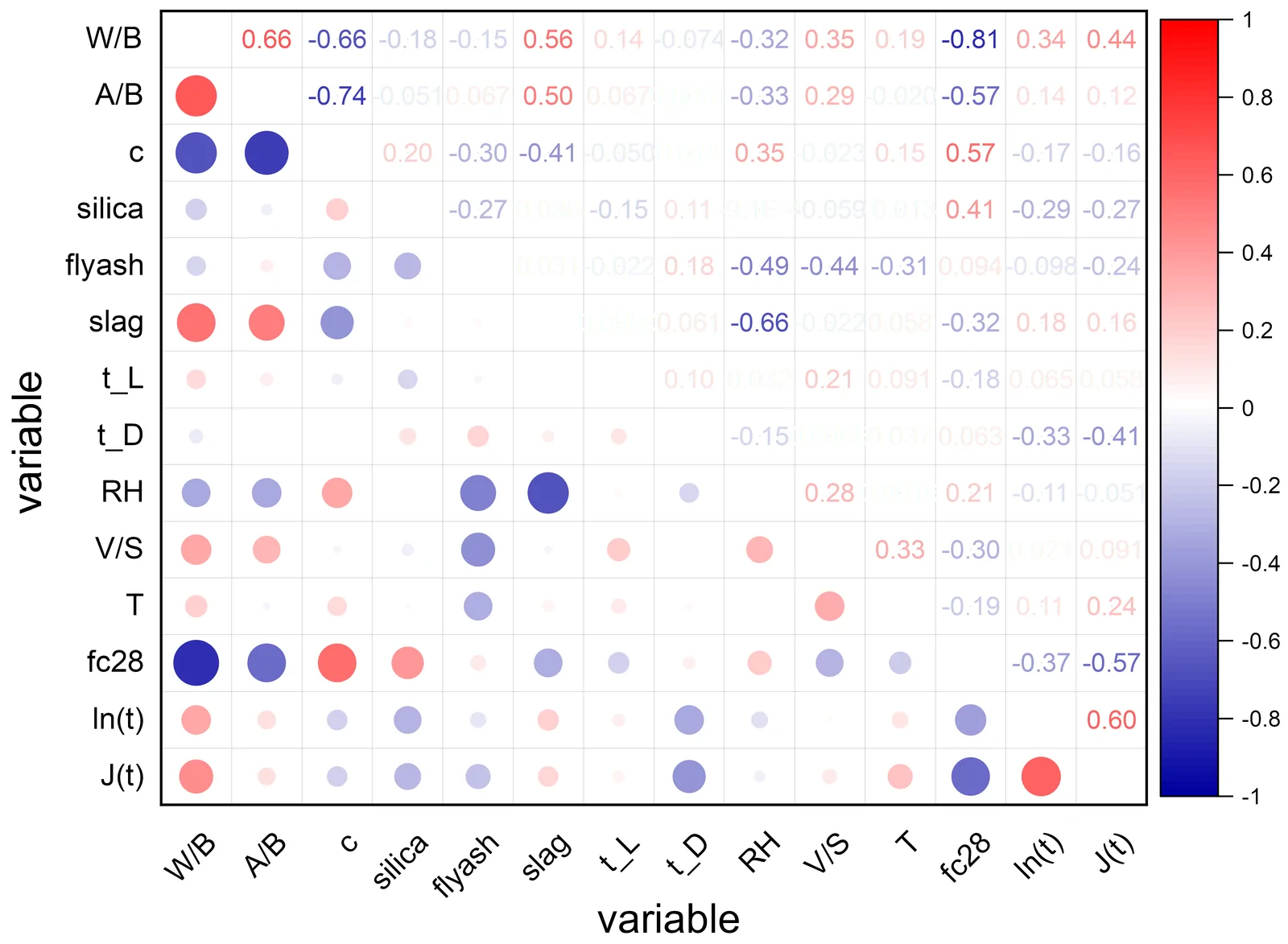
Heatmap of input correlation coefficients.

Figure 1 presents a correlation heatmap showing the linear associations between input variables and between each input variable and creep compliance J(t). In the heatmap, bubble area and color in the lower triangle represent the strength and sign of the Pearson correlation coefficients, while the upper triangle gives the corresponding numerical values. For the relationships between input variables and the target variable, the logarithm of loading duration ln(t) was moderately positively correlated with J(t) (r=0.59), indicating that creep compliance increased continuously with loading duration, consistent with the fundamental time-dependent behavior of concrete creep. The 28-day compressive strength $f_{c28}$ was moderately negatively correlated with J(t) (r=−0.51), indicating that a higher matrix strength and denser microstructure corresponded to lower creep compliance. Age at loading $t_{D}$ was moderately negatively correlated with J(t) (r=−0.46), indicating that greater concrete maturity at loading resulted in less subsequent creep development. The water-to-binder ratio W/B was weakly to moderately positively correlated with J(t) (r=0.39); increasing W/B increased the porosity and interfacial transition zone defects of the hardened cement paste, thereby promoting creep development. In contrast, the remaining variables, including cement content c, silica fume content, fly ash content, slag content, volume-to-surface area ratio V/S, ambient temperature T, and curing age t_L, had absolute correlation coefficients with J(t) below 0.30, indicating weak independent linear effects. Several groups of moderately to highly correlated input variables were also present: W/B was strongly negatively correlated with the 28-day compressive strength $f_{c28}$ (r=−0.81), and the aggregate-to-binder ratio A/B was relatively strongly negatively correlated with cement content c (r=−0.73). In addition, W/B and A/B (r=0.61), A/B and $f_{c28}$ (r=−0.54), fly ash and relative humidity RH (r=−0.55), and slag and RH (r=−0.52) reached moderate or stronger correlations. These results indicate the presence of multicollinearity that warrants attention; however, most of these relationships arise from physical derivation or coordinated variation among mix proportion parameters. Mechanically removing variables solely on the basis of correlation coefficients may therefore weaken the physical completeness and interpretability of the model. Accordingly, Figure 1 was used mainly to identify risks of variable redundancy, and the final feature set was determined by comprehensively considering creep mechanisms, the theoretical requirements of the B4 model, and previous studies.

To further examine multicollinearity among input features, variance inflation factors (VIFs) were calculated based on the training set, with the complete results listed in Table 2. All VIFs were below 10, with a maximum of 6.46, indicating that although some variables were correlated, severe multicollinearity was absent.

Based on the above considerations, the input features were selected to ensure the generalization ability and physical consistency of the prediction model. The theoretical framework of the B4 creep model indicates that concrete compressive strength is one of the core material parameters affecting creep because it comprehensively reflects the effects of the water-to-binder ratio, degree of cement hydration, and aggregate characteristics on microstructural creep mechanisms [19]. Therefore, although Figure 1 shows strong correlations among some variables, directly removing them would weaken the physical completeness of the B4 model and could discard important information related to creep mechanisms. On this basis, the feature design strategy of Liang et al. [33], who used supervised machine learning algorithms to predict the high-temperature creep of concrete, was considered. Because compressive strength and elastic modulus are strongly correlated as material properties, as both originate from the evolution of the matrix microstructure, elastic modulus was not included as an independent variable; instead, the 28-day standard-cured compressive strength was used to indirectly represent the stiffness characteristics of the matrix. Considering the physical mechanisms, data availability, and the requirements of subsequent multi-objective optimization, 14 input features were ultimately selected, covering material composition, loading and curing conditions, and environmental conditions: water-to-binder ratio, aggregate-to-binder ratio, cement content, cement type, silica fume replacement ratio, fly ash replacement ratio, slag replacement ratio, age at loading, drying age, relative humidity, volume-to-surface area ratio (V/S), ambient temperature, 28-day compressive strength, and loading duration. Cement type is a categorical variable. According to ASTM C150 and the B4 model, cement type was classified as normal-setting (R), rapid-setting (RS), or slow-setting (SL). Table 3 describes the correspondence between the three cement types and their numerical codes, and the encoded variables were included with the other continuous features in the model input matrix. To avoid treating cement type as an ordinal variable, R, RS, and SL were uniformly converted into three binary indicator variables before training. RF, XGBoost, BPNN, SVGP, and the XGBoost strength surrogate used in the optimization stage all adopted this one-hot encoding. During model training and testing, fc28 was taken directly from the experimental database; in the subsequent NSGA-II optimization stage, candidate mix proportions do not have measured strength values, so fc28 was predicted by a separately trained and validated XGBoost strength surrogate. The detailed mapping is provided in Section 7.1.

### 2.4. Physical-Prior-Assisted Residual Learning Strategy

**Figure 2:**
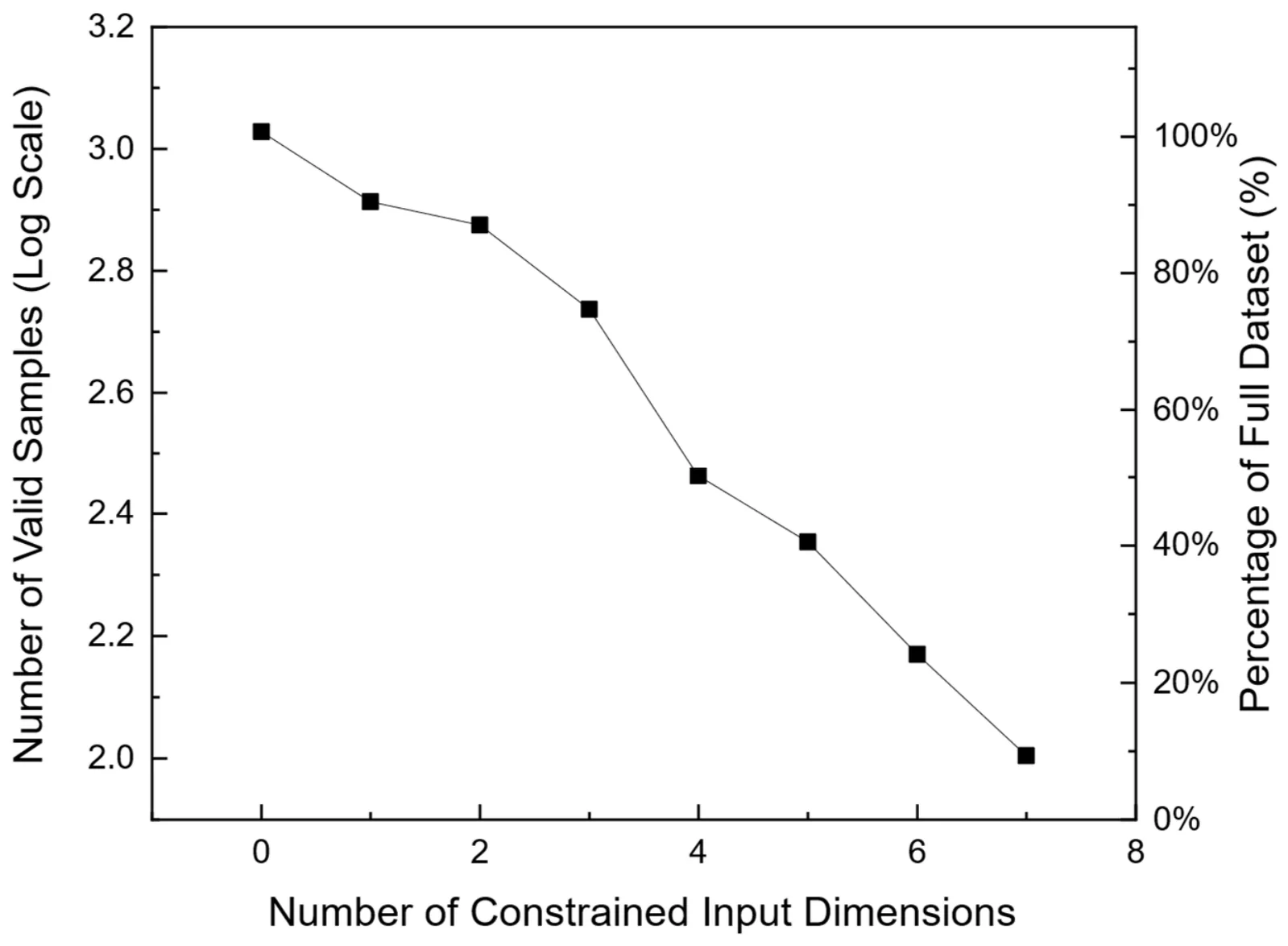
Relationship between dimensionality and sample size.

Experimental studies on the creep of low-carbon concrete remain insufficient, and data acquisition is time-consuming and costly, resulting in a limited number of available samples. In addition, during multi-objective optimization for practical engineering, environmental variables such as relative humidity and temperature must be fixed at constant values according to the target service conditions and cannot be treated as optimization variables. This further reduces the dimensionality of the input features. Once the environmental variables are fixed, the number of samples available for model training decreases sharply; as shown in Figure 2, the retained data account for only 10.2% of the full dataset after compression across multiple dimensions. Purely data-driven models are therefore highly prone to overfitting. SVGP, as a nonparametric Bayesian method, has a kernel prior whose smoothness assumption matches the small-amplitude and smooth creep-residual surface and provides small-sample learning capability [24], making it particularly suitable for capturing complex nonlinear patterns that the physical model cannot describe under sparse data conditions. Modeling the systematic bias of a physical model using a Gaussian process is a mature paradigm [34], and sparse variational inference provides a scalable approximation for medium-scale datasets [35,36]. In addition, the predictive uncertainty of SVGP offers a natural diagnostic for evaluating the reliability of the surrogate model within the optimization domain. By treating the B4 prediction as the backbone output, the SVGP model learns the residual. The final creep prediction is expressed as follows:

(2) $$\begin{array}{c} \varepsilon_{\mathrm{cr}}=\varepsilon_{\mathrm{cr},B4}(X)+\hat{∆}\varepsilon_{\mathrm{cr}}(X) \end{array}$$

**Figure 3:**
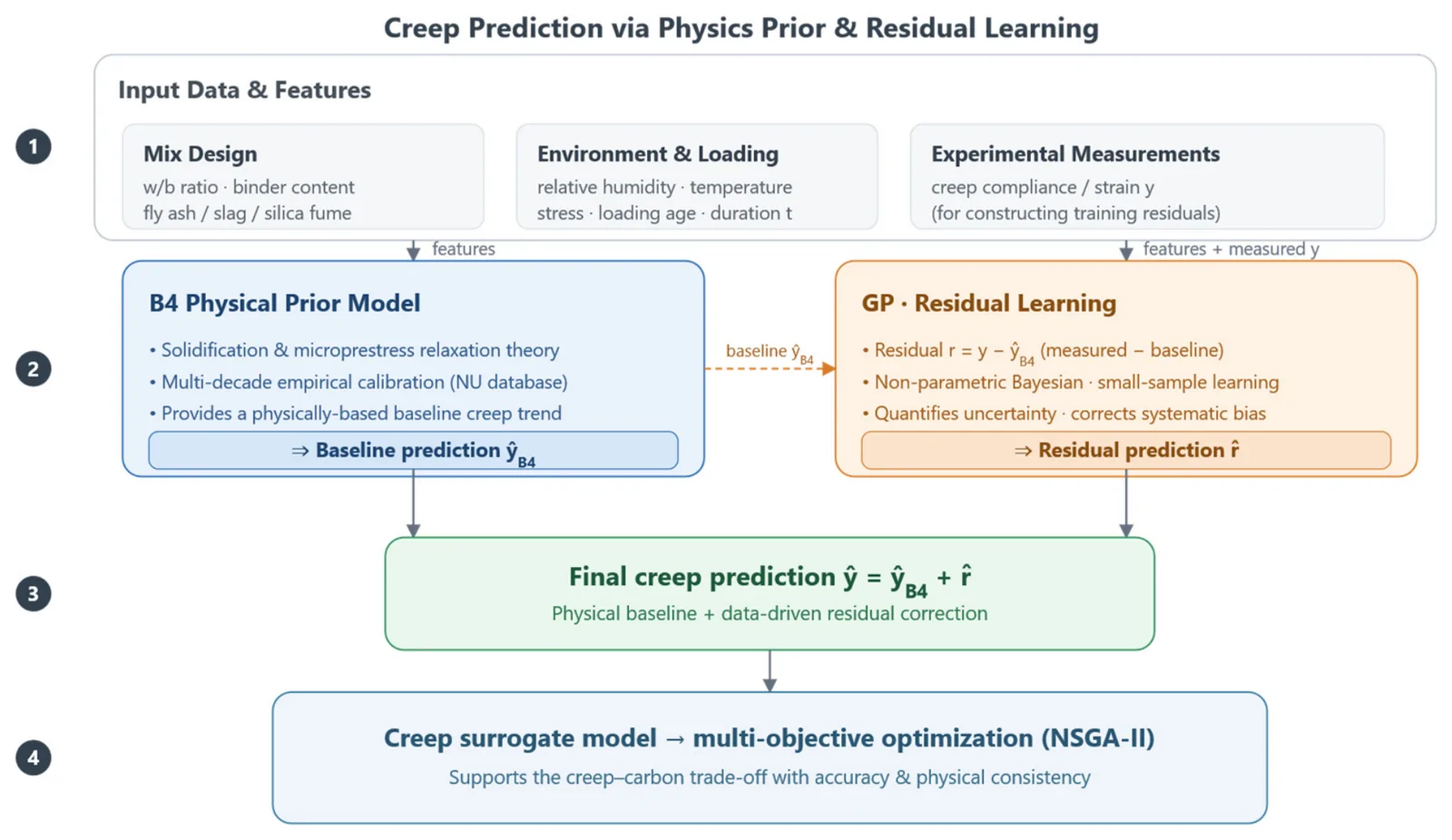
Schematic of the modeling workflow for physical prior guidance and residual learning.

This framework retains the ability of traditional creep theory to describe the main creep trend and uses a data-driven method to correct the systematic bias of the empirical model for actual materials. It therefore stably improves prediction accuracy under limited training data and provides a reliable surrogate model for subsequent multi-objective optimization of creep and carbon emissions in low-carbon concrete. The overall modeling workflow is shown in Figure 3.

### 2.5. Construction of the B4 Creep Prediction Model

The B4 creep model was systematically recalibrated and extended by the Bažant research team from model B3, the method recommended by RILEM in 1995 [19,37]. Based on solidification theory and microprestress relaxation theory, it provides reasonable functional forms for the evolution of creep and shrinkage over multiple time scales [20,21]. Compared with empirical models such as ACI 209, the CEB-fib Model Code, and the Gardner-Lockman (GL) model, B4 explicitly accounts for mix proportion factors such as binder type, mineral admixtures, chemical admixtures, and aggregate type, and separately represents drying shrinkage and autogenous shrinkage, making it better suited to low-carbon concrete. Its parameters were calibrated using the NU database and long-term deflection data from long-span prestressed box-girder bridges, providing reliable extrapolation over multi-decade time scales [37,38]. Therefore, this study uses the B4 model as the physical prior baseline to constrain subsequent residual learning and provide a baseline for the creep constraints in multi-objective optimization.

Within the normal service stress range of concrete, in which the stress level does not exceed 0.4 times the mean cylinder compressive strength ${\bar{f}}_{c}$, creep can be treated as a linear superposition. At age t′, under a constant uniaxial compressive stress σ, the total strain at any time t is given by:

(3) $$\begin{array}{c} \varepsilon (t)=J(\hat{t},\hat{t}\prime )\cdot \sigma +\varepsilon_{sh,\mathrm{total}}(\tilde{t},{\tilde{t}}_{0})+\alpha_{T}∆T \end{array}$$

where $J(\hat{t},\hat{t}\prime )$ is the creep compliance function; after a unit constant stress is applied at age t′, it is defined as the total strain produced at time t, including instantaneous elastic strain and creep strain; σ is the constant uniaxial compressive stress (positive in tension); $\varepsilon_{sh,\mathrm{total}}(\tilde{t},{\tilde{t}}_{0})$ is the stress-free shrinkage strain; $\alpha_{T}$ is the coefficient of thermal expansion and ∆T is the temperature change; $\hat{t}$ and $\hat{t}\prime$ are the temperature-corrected current and loading equivalent times, and $\tilde{t}$ and ${\tilde{t}}_{0}$ are the temperature-corrected drying age and initial drying age.

The above expression divides total strain into stress-dependent and stress-independent terms. The former is driven by sustained stress and can be represented by $J(\hat{t},\hat{t}\prime )\cdot \sigma$, including instantaneous elastic deformation and time-dependent creep; the latter is caused by shrinkage and temperature changes and can be represented by $\varepsilon_{sh,\mathrm{total}}(\tilde{t},{\tilde{t}}_{0})+\alpha_{T}∆T$. Accordingly, B4 parameterizes the individual deformation components independently to preserve the interpretability of creep and shrinkage mechanisms [19].

B4 decomposes the total compliance function $J(\hat{t},\hat{t}\prime )$ into the sum of instantaneous compliance, basic creep compliance, and drying creep compliance:

(4) $$\begin{array}{c} J(\hat{t},\hat{t}\prime )=q_{1}+C_{0}(\hat{t},\hat{t}\prime )+C_{d}(\hat{t},\hat{t}\prime ,{\tilde{t}}_{0}) \end{array}$$

where $q_{1}$ is the instantaneous compliance ($q_{1}=p_{1}/E_{28}$), $p_{1}$ is a dimensionless empirical coefficient related to cement type, and $E_{28}$ is the elastic modulus at 28 days; this term represents the recoverable deformation immediately after loading and can be approximated as independent of the age at loading [19]. The basic creep compliance $C_{0}(\hat{t},\hat{t}\prime )$ describes the long-term viscoelastic deformation of concrete under sealed conditions, and its rate form is derived from solidification theory [20]:

(5) $$\begin{array}{c} {\dot{C}}_{0}(t,t\prime )=\frac{n(q_{2}t^{-m}+q_{3})}{(t-t\prime )+(t-t\prime {)}^{1-n}}+\frac{q_{4}}{t}\;\;\;(m=0.5,\;n=0.1) \end{array}$$

The rate equation consists of two parts: Here, controls aging viscoelastic creep, and controls non-aging viscoelastic creep. The second term represents irreversible flow creep, whose rate is inversely proportional to the current age and exhibits unbounded growth on the scale, which is key to the multi-decade extrapolation capability of B4 [19,21]. The drying creep compliance characterizes the Pickett effect caused by coupling between moisture diffusion and the stress field; its magnitude depends on the difference between the mean pore humidity functions at the current time and the loading start time, respectively [19]:

(6) $$\begin{array}{c} C_{d}(\hat{t},\hat{t}\prime ,{\tilde{t}}_{0})=q_{5}{\left[\exp\left(-p_{5H}H(\hat{t},{\tilde{t}}_{0})\right)-\exp\left(-p_{5H}H_{c}({\hat{t}}_{0}\prime ,{\tilde{t}}_{0})\right)\right]}^{0.5} \end{array}$$

The pore humidity function uses $s_{sh}$ as the characteristic time and approaches the environmental humidity h through a hyperbolic tangent form:

(7) $$\begin{array}{c} H(\hat{t},{\tilde{t}}_{0})=1-(1-h)\tan h\sqrt{\frac{\hat{t}-{\tilde{t}}_{0}}{s_{sh}}} \end{array}$$

The creep parameters $q_{2}$–$q_{5}$ are determined by base coefficients related to cement type $p_{2},p_{3},p_{4},p_{5}$ and power-law relationships with W/B and A/C; their complete forms are given in reference [19]. When materials such as fly ash, silica fume, and superplasticizers are incorporated, the model modifies $q_{2}$–$q_{5}$ using category-specific multiplier coefficients to reflect the independent effects of each component on the creep components [19].

The total shrinkage strain $\varepsilon_{sh,\mathrm{total}}$ is obtained by superimposing drying shrinkage $\varepsilon_{sh}$ and autogenous shrinkage $\varepsilon_{\mathrm{au}}$:

(8) $$\begin{array}{c} \varepsilon_{sh,\mathrm{total}}(\tilde{t},{\tilde{t}}_{0})=\varepsilon_{sh}(\tilde{t},{\tilde{t}}_{0})+\varepsilon_{\mathrm{au}}(\tilde{t},{\tilde{t}}_{0}) \end{array}$$

Drying shrinkage begins at the exposure time $t_{0}$, and its evolution follows a hyperbolic tangent form derived from diffusion theory:

(9) $$\begin{array}{c} \varepsilon_{sh}(\tilde{t},{\tilde{t}}_{0})=\varepsilon_{sh,\infty }({\tilde{t}}_{0})\cdot k_{h}\cdot \tan h\sqrt{\frac{\tilde{t}}{s_{sh}}} \end{array}$$

where $\varepsilon_{sh,\infty }({\tilde{t}}_{0})$ is the ultimate drying shrinkage corrected for aging effects, $k_{h}=1-h^{3}$ (h<0.98) is the environmental humidity correction factor, and $s_{sh}$ is the drying shrinkage half-life $s_{sh}=s_{0}\cdot k_{\mathrm{sa}}\cdot (k_{s}\cdot D{)}^{2}(D=2V/S$), where $k_{s}$ is the section shape factor and $k_{\mathrm{sa}}$ is the aggregate type correction factor; the influence of $D^{2}$ on the half-life is consistent with the moisture diffusion equation [19,39]. Autogenous shrinkage $\varepsilon_{\mathrm{au}}$ begins at the final setting time t=0 and results from the combined effects of chemical shrinkage and self-desiccation; it cannot be neglected under low water-to-binder ratios (W/C<0.40). B4 uses an S-shaped function to describe its evolution:

(10) $$\begin{array}{c} \varepsilon_{\mathrm{au}}(\tilde{t},{\tilde{t}}_{0})=\varepsilon_{\mathrm{au},\infty }{\left[1+{\left(\frac{s_{\mathrm{au}}}{\tilde{t}+{\tilde{t}}_{0}}\right)}^{\alpha }\right]}^{r_{t}} \end{array}$$

where $\alpha =r_{a}(w/c/0.38)$; the ultimate autogenous shrinkage $\varepsilon_{\mathrm{au},\infty }$, half-life $s_{\mathrm{au}}$, and exponents $r_{t}$ and $r_{a}$ are determined by the cement type and admixture correction coefficients [19].

The experimental data in this study were obtained under constant temperature within each test group, and the set temperature among groups was T=20∼25 °C. Because the temperature remained constant within each test group, ∆T=0; consequently, the thermal expansion contribution $\alpha_{T}∆T$ was neglected. However, isothermal conditions do not mean that temperature does not control the deformation rate; hydration, moisture diffusion, and viscous flow of the gel are thermally activated processes. Based on the Arrhenius equation, B4 multiplies the actual time by $b_{T}$ and maps it to an equivalent time at a reference temperature of 20 °C [19,40]:

(11) $$\begin{array}{c} b_{T}=\exp\left[\frac{U}{R}\left(\frac{1}{293}-\frac{1}{T+273}\right)\right] \end{array}$$

where U/R is the ratio of activation energy to the gas constant, with U/R=4000 K used when data are unavailable. B4 further defines hydration, drying, and creep equivalent-time factors $b_{Th}$, $b_{\mathrm{Ts}}$, and $b_{\mathrm{Tc}}$ to correct the aging, drying, and creep rates, respectively [19].

## 3. Construction of the Residual Learning Model Based on a Gaussian Process

### 3.1. Residual Learning Framework and Global Linear Calibration

The B4 creep model is based on solidification theory and has been calibrated with extensive experimental data. It inherently incorporates macroscopic physical laws, including the monotonicity and limiting behavior of creep evolution with respect to the water-to-binder ratio, age at loading, and environmental humidity. However, when the model is applied to new low-carbon concrete mixes, systematic deviations between predicted and measured values often occur because of differences in material composition and microstructure. If Gaussian process regression (GP) is directly used to model the absolute creep compliance $J_{o\mathrm{bs}}(x,t)$, the model may violate known physical priors in data-sparse regions and produce nonmonotonic or unreasonable predictions. To address this issue, this study adopts a residual learning framework in which the backbone prediction provided by the B4 model is treated as a physical prior baseline and only the correction between experimental observations and the physical model is learned in a data-driven manner.

Let the input feature vector be x, including material parameters, age at loading, environmental conditions, and loading duration. The theoretical creep compliance given by the B4 model is $J_{B4}(x,t)$. To eliminate the systematic offset that the B4 model may exhibit over the entire dataset, global linear calibration was introduced before residual learning. Its specific form is as follows:

(12) $$\begin{array}{c} {\underset{\alpha ,\beta }{\min}\sum_{i=1}^{N}\left(J_{\mathrm{obs}}^{\left(i\right)}-\alpha J_{B4}^{\left(i\right)}-\beta \right)}^{2} \end{array}$$

where $J_{o\mathrm{bs}}^{\left(i\right)}$ is the measured creep compliance of the ith sample and (α,β) are the global calibration coefficients obtained by least-squares linear regression over all samples in the training set. After calibration, the physical model output is $\alpha J_{B4}(x,t)+\beta$, and the residual label is defined as follows:

(13) $$\begin{array}{c} {∆}^{\left(i\right)}=J_{\mathrm{obs}}^{\left(i\right)}-\left(\begin{array}{c} \alpha J_{B4}^{\left(i\right)}+\beta \end{array}\right) \end{array}$$

The task of the GP is thereby transformed into regression on the residual ∆(x). The final predicted creep compliance is expressed as follows:

(14) $$\begin{array}{c} \hat{J}(x,t)=\alpha J_{B4}(x,t)+\beta +{∆}_{GP}(x) \end{array}$$

Note that B4 serves as the backbone prior, whereas neither the linear calibration nor the SVGP residual imposes a hard constraint; therefore, the final model is not guaranteed to strictly preserve the monotonicity, positivity, or asymptotic behavior of B4. The role of the B4 backbone is to provide a trend prior and reduce the risk of implausible extrapolation in sparse regions, rather than to formally exclude all nonphysical predictions; the applicability of the final model still needs to be evaluated together with parameter-domain coverage and error diagnostics. Within this framework, the SVGP prediction target is reduced from the entire highly nonlinear compliance surface to a residual surface with smaller amplitude and relatively smooth variation, substantially reducing dependence on the number of training samples and helping lower the risk of implausible predictions in sparse regions under scarce creep data. The resulting linear calibration coefficients are serialized and saved after training and loaded during inference, ensuring consistency of the prediction workflow.

### 3.2. Sparse Variational Gaussian Process Model Architecture

#### 3.2.1. Sparse Approximation and Variational Inference

Exact Gaussian process regression has a time complexity of $O(N^{3})$, where N is the number of training samples. To reduce computational cost, this study uses a sparse variational Gaussian process (SVGP) for residual modeling [41].

SVGP introduces a set of M inducing points, $Z=z_{m}{\}}_{m=1}^{M}$, and defines the inducing variables u=f(Z). The evidence lower bound (ELBO) of the log marginal likelihood is optimized as follows:

(15) $$\begin{array}{c} L_{E\mathrm{LBO}}={\sum_{i=1}^{N}E}_{q(u)}[\log p({∆}^{\left(i\right)}|f(x_{i}))]-KL[q(u)\|p(u)] \end{array}$$

where the variational distribution q(u)=N(m,S) is parameterized by the mean m and covariance S at the inducing points. This method reduces the computational complexity to $O(NM^{2})$ and can efficiently handle medium-scale datasets while preserving approximation quality.

#### 3.2.2. Selection of the Mean Function and Covariance Kernel

Although the residual ∆ has a relatively small overall amplitude, it may still exhibit a first-order linear trend caused by variations in key parameters such as the water-to-binder ratio and 28-day strength. To absorb this effect, the mean function was set to a linear mean rather than the default constant zero mean:

(16) $$\begin{array}{c} \mu (x)=c^{⊤}x+b \end{array}$$

The linear mean allows the covariance kernel to focus on capturing the remaining second-order nonlinear patterns, thereby effectively improving the approximation capability of the model. The covariance function uses a Matérn-5/2 kernel with automatic relevance determination (ARD), nested within a scale kernel to adaptively adjust the overall variance:

(17) $$\begin{array}{c} k(x,x\prime )=\sigma_{f}^{2}\cdot \left(1+\sqrt{5}\;r+\frac{5}{3}r^{2}\right)\exp\left(-\sqrt{5}\;r\right),\;r=\sqrt{\sum_{d=1}^{D}\frac{(x_{d}-x_{d}\prime {)}^{2}}{l_{d}^{2}}} \end{array}$$

where $\sigma_{f}^{2}$ is the output scale and $l_{d}$ is the length scale of the dth input dimension. The Matérn-5/2 kernel corresponds to sample paths that are twice differentiable, which agrees with the expected smoothness of the concrete creep residual surface. Compared with the infinitely differentiable radial basis function (RBF) kernel, it better represents local details and is therefore more consistent with the nonsmooth characteristics of physical systems. The ARD mechanism assigns an independent length scale to each input dimension, enabling the model to automatically distinguish the strength of the influence of each physical factor on the residual and enhancing interpretability.

#### 3.2.3. Regularization Strategy Against Kernel Collapse

In high-dimensional regression under small-sample conditions, the kernel of a GP model is highly susceptible to kernel collapse during optimization of the evidence lower bound, where the length scale tends to zero. When the length scale of a dimension satisfies $l_{d}\to 0$, the covariance matrix degenerates to white noise, and the model completely loses generalization capability in that dimension. To avoid this problem, this study adopts two regularization measures:

Length-scale lower bound: A hard lower bound $l_{d}\geq 0.1$ was imposed on the length scale of each dimension in the ARD kernel using a GreaterThan(0.1) transformation, numerically preventing degeneration in any dimension.

Length-scale prior: Within the Bayesian framework, a Gamma prior distribution $l_{d}∼\Gamma \left(2,2\right)$ was assigned to the length scale. Its mode is 0.5, and the probability density provides a flexible constraint on (0.1,∞). This prior encourages learning moderate-scale correlations and suppresses overfitted length-scale estimates caused by data sparsity.

### 3.3. SVGP Training Settings and Initialization Strategy

The SVGP model was trained with the Adam optimizer (learning rate 0.01) by maximizing the evidence lower bound (ELBO) in full-batch mode, with a maximum of 300 training epochs and an ELBO-based early-stopping rule (patience = 40). Model parameters were initialized as follows: 200 inducing points were first initialized by K-means clustering on the normalized training inputs and then jointly learned with the remaining model parameters; the variational posterior used a whitened parameterization with its mean initialized to zero; the ARD kernel length scales were initialized to 1.0 in each input dimension, remained learnable during training, and were subject to the lower-bound constraint described in Section 3.2.3 to prevent kernel collapse; the weights of the linear mean function were initialized by least-squares estimation using the training-fold data. These training settings and initialization strategies were kept identical across all cross-validation folds.

## 4. Calculation of Carbon Emissions for Low-Carbon Concrete

### 4.1. System Boundary and Assessment Time for Net Carbon Emissions

Environmental impact assessment of concrete structures must cover the complete life cycle from raw material acquisition and production and transportation to the use phase [42]. Because transportation routes, energy structures, and other factors involving external supply chains and site-specific conditions are difficult to treat as unified decision variables within the mix proportion optimization scope of this study, the system boundary was limited to the production stage, and the implications of this boundary choice are discussed below. However, the uptake of atmospheric CO_2_ by concrete through carbonation during the use phase, that is, the carbon sink effect, cannot be ignored, particularly for accurate assessment of the long-term carbon balance of low-carbon concrete [43]. Therefore, this study considers the carbon balance in terms of both emissions and uptake and defines the net carbon emissions per unit volume of concrete, as follows:

(18) $$\begin{array}{c} E_{\mathrm{net}}=E_{\mathrm{prod}}-E_{\mathrm{carb}} \end{array}$$

where is the carbon emissions generated during raw material production and transportation and is the amount of CO_2_ absorbed by carbonation during the service life of concrete. The baseline assessment time in Equation (18) was strictly aligned with the assessment time of the creep prediction model, extending from the loading start time to the 1000-day baseline. This ensures that carbon emissions and creep performance are comparable in the time dimension during multi-objective optimization.

### 4.2. Carbon Emission Calculation

Production-stage carbon emissions originate from the extraction, processing, and transportation of the raw materials used in concrete. For concrete per unit volume, production emissions are obtained by summing the products of the mass of each component and its corresponding carbon emission factor:

(19) $$\begin{array}{c} E_{\mathrm{prod}}=\sum_{i}m_{i}\cdot EF_{i} \end{array}$$

where is the amount of the raw material per unit volume of concrete (kg/m^3^) and is its carbon emission factor (kg CO_2_e/kg). The carbon emission factors used in this study were mainly obtained from domestic and international life cycle assessment (LCA) databases and related studies [44,45,46], and the specific values are listed in Table 4. The emission factors in Table 4 are representative values. Because regional energy structures, transportation distances, and production technologies may alter these factors, local LCA databases can be used to update them in practical applications.

During the service life of concrete, atmospheric CO_2_ diffuses through the pore network and dissolves in pore water, reacting with cement hydration products, mainly calcium hydroxide and calcium silicate hydrate, to form calcium carbonate through carbonation [47]. This process partially offsets production-stage carbon emissions and constitutes the carbon sink of concrete. Accurate simulation of carbonation requires solving the coupled diffusion-reaction equations for CO_2_ in unsaturated porous media, which is computationally complex and depends on numerous microscopic parameters. To balance computational efficiency and mechanistic rationality in multi-objective optimization, this study adopts a simplified engineering model based on classical carbonation theory.

The classical carbonation depth model gives a square-root relationship between carbonation depth and time, that is, [47,48]. Assuming that the total amount of CO_2_ absorbed by carbonation is proportional to the carbonation depth, the carbonation uptake also follows a square-root scaling law with time. Based on studies by Pade et al. [43] and Possan et al. [49] on the life-cycle carbon uptake of ordinary concrete, carbonation uptake for concrete with medium permeability can reach 15–30% of production emissions over a period of 50–100 years. At shorter ages, such as 1000 days, the carbonation uptake decreases substantially to approximately 3% of production emissions. Accordingly, this study sets the baseline carbonation uptake ratio as follows: at days, the carbonation uptake is equal to 3% of production-stage emissions. It should be noted that this carbonation treatment is an indicative simplification [43,49]. Under the assumption that all candidate mix proportions use the same carbonation uptake ratio, varying this ratio uniformly from 0 to 6% only rescales the net carbon emissions proportionally and does not change the Pareto dominance relationships or TOPSIS ranking. This conclusion applies specifically to the proportional assumption; if different mix proportions have different carbonation uptake ratios because of differences in pore structure, cementitious composition, or service environment, the Pareto front and TOPSIS ranking may change.

## 5. Model Selection and Prediction Performance Comparison

**Figure 4:**
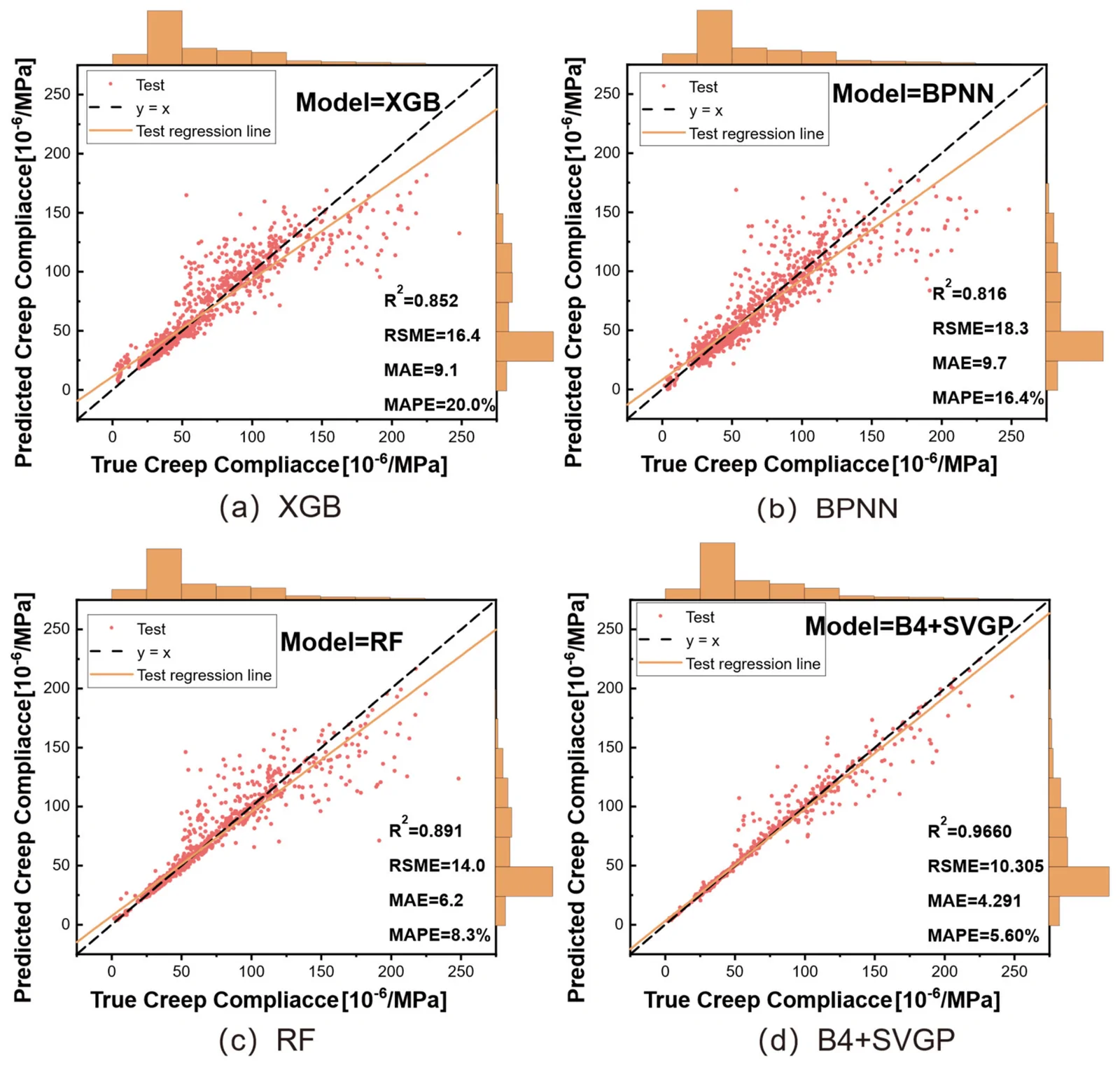
Scatter plots of different models for predicting creep compliance of low-carbon concrete.

The reliability of the multi-objective optimization results depends on the prediction accuracy of the creep surrogate model. Therefore, under a unified dataset, feature set, and training protocol, this study compares B4+SVGP with three representative data-driven models: random forest (RF), extreme gradient boosting (XGBoost), and a backpropagation neural network (BPNN), which represent ensemble learning, gradient boosting, and neural-network paradigms, respectively. RF reduces variance through Bagging sampling and averaging over multiple trees and is robust to noise and outliers [50]. XGBoost sequentially adds weak learners along the residual direction and suppresses overfitting through L1/L2 regularization, column sampling, and learning-rate shrinkage [51]. BPNN approximates nonlinear mappings through error backpropagation but may exhibit either overfitting or underfitting risk when parameter scale and sample size are mismatched [52]. The four models were constructed under exactly the same experimental protocol: the 14 input features were uniformly normalized using min-max scaling, the output creep compliance was normalized synchronously, and predictions were inverse-transformed to the original scale. All performance metrics were obtained through 10-fold cross-validation: 1066 samples were divided into 10 mutually exclusive folds, with six test folds containing 107 samples each and four test folds containing 106 samples each; the corresponding training folds contained 959 or 960 samples, giving a training/test ratio of approximately 9:1 in each fold. All four models used identical fold assignments, and each sample was used once as a test sample. Fold assignments were generated with a fixed random seed to ensure comparability and reproducibility. Fold generation used a random seed of 42, and a common training random seed of 56 was used for all compared models to control training randomness within each fold. Input and output normalization parameters were fitted only on the training folds, and hyperparameter searches for the baseline models (RF, XGBoost, and BPNN) were also performed only within the training folds. B4+SVGP used the number of inducing points, learning rate, number of training epochs, and early-stopping strategy specified in Section 3.3 and Table 5. The final model performance was reported as the mean ± standard deviation of the test metrics across folds. It should be noted that the merged database did not retain a unified experimental-series identifier or source-group field; therefore, the current fold assignment is record-level. The folds are mutually exclusive, but correlated records from the same mix proportion or publication source may still occur in different folds. Therefore, the current 10-fold cross-validation primarily evaluates record-level out-of-fold prediction performance, and the reported results should be interpreted within this validation scope; extrapolation to independently sampled mix proportions should be treated with caution. Because all models use the same folds and all preprocessing and tuning are confined to the training folds, these comparisons provide an internally consistent benchmark under one record-level protocol, and applicability at the independent mixture level can be examined in future work. This setup maintains model comparability and reproducibility without introducing additional data assumptions; the cross-fold distribution of correlated records mainly affects the external interpretation of the accuracy estimate and does not alter the internal consistency of the relative comparison under the same record-level protocol. Once experimental-series or source-group identifiers become available, grouped validation can be added within the existing framework to further examine the robustness of the conclusions. The hyperparameter optimization results for each model are listed in Table 5. The BPNN architecture (16-128-64-32-1) and number of training epochs were determined by a candidate-architecture search within the training folds: three hidden layers (128-64-32) provided a favorable balance between validation accuracy and parameter count, whereas deeper or wider architectures did not provide stable improvements. The input dimension of 16 corresponds to the 14 features after one-hot expansion of cement type. The batch sizes and early-stopping rules are also listed in Table 5. The scatter plots in Figure 4 are the aggregated out-of-fold predictions from the 10 test folds. The BPNN used here is only a feedforward neural-network baseline for tabular data and is not treated as a deep-learning model; its architecture and training settings are provided in Table 5.

This study evaluates model performance comprehensively using the coefficient of determination (R^2^), root mean square error (RMSE), mean absolute error (MAE), and mean absolute percentage error (MAPE). Let the number of test samples in each fold be n, and let the measured creep compliance, predicted value, and mean measured value of the ith sample be y_i_, ŷ_i_, and ȳ, respectively. Each metric was calculated within each test fold and reported as the 10-fold mean ± standard deviation. The metrics are defined as follows:

(20) $$\begin{array}{c} R^{2}=1-\frac{\sum_{i}(y_{i}-{\hat{y}}_{i}{)}^{2}}{\sum_{i}(y_{i}-\bar{y}{)}^{2}} \end{array}$$

(21) $$\begin{array}{c} RMSE=\sqrt{\frac{1}{n}\sum_{i}(y_{i}-{\hat{y}}_{i}{)}^{2}} \end{array}$$

(22) $$\begin{array}{c} MAE=\frac{1}{n}\sum_{i}|y_{i}-{\hat{y}}_{i}| \end{array}$$

(23) $$\begin{array}{c} MAPE=\frac{100\%}{n}\sum_{i}\frac{\left|y_{i}-{\hat{y}}_{i}\right|}{y_{i}} \end{array}$$

where R^2^ reflects the degree to which the model explains the measured variance; RMSE and MAE measure absolute errors, with RMSE being more sensitive to outliers and MAE being relatively robust; and MAPE measures relative error but is easily amplified by samples close to zero. Because multi-objective optimization directly coordinates the absolute creep compliance with the carbon emission objective, RMSE and MAE were used as the main evaluation criteria, and MAPE was used only as a supplementary reference.

Figure 4 presents scatter plots of the out-of-fold predicted versus measured values for the four models obtained by 10-fold cross-validation. Under record-level validation, B4+SVGP achieved the best average values for all four metrics: R^2^ reached 0.9660, RMSE was 10.305 × 10^−6^/MPa, MAE was 4.291 × 10^−6^/MPa, and MAPE was only 5.60%. The predicted points were closely distributed around the y = x line, with only slight dispersion in the region of higher creep compliance. RF showed the second-best performance (R^2^ = 0.891), and its points generally followed the trend line, although the overall error increased noticeably. The fitting accuracy of XGBoost and BPNN decreased further. In particular, the R^2^ of BPNN decreased to 0.816 and its RMSE increased to 18.3 × 10^−6^/MPa, with evident systematic deviation in the high-compliance region. It should be noted that although the RMSE and MAE of XGBoost were slightly lower than those of BPNN, its MAPE was as high as 20.0%, clearly exceeding the 16.4% of BPNN. This indicates that XGBoost produced large relative errors for some creep samples with small values and that MAPE was significantly affected by the amplification effect of near-zero samples. This phenomenon suggests that relying solely on MAPE may underestimate the absolute accuracy of a model and highlights the necessity of using RMSE and MAE as the core evaluation metrics.

**Figure 5:**
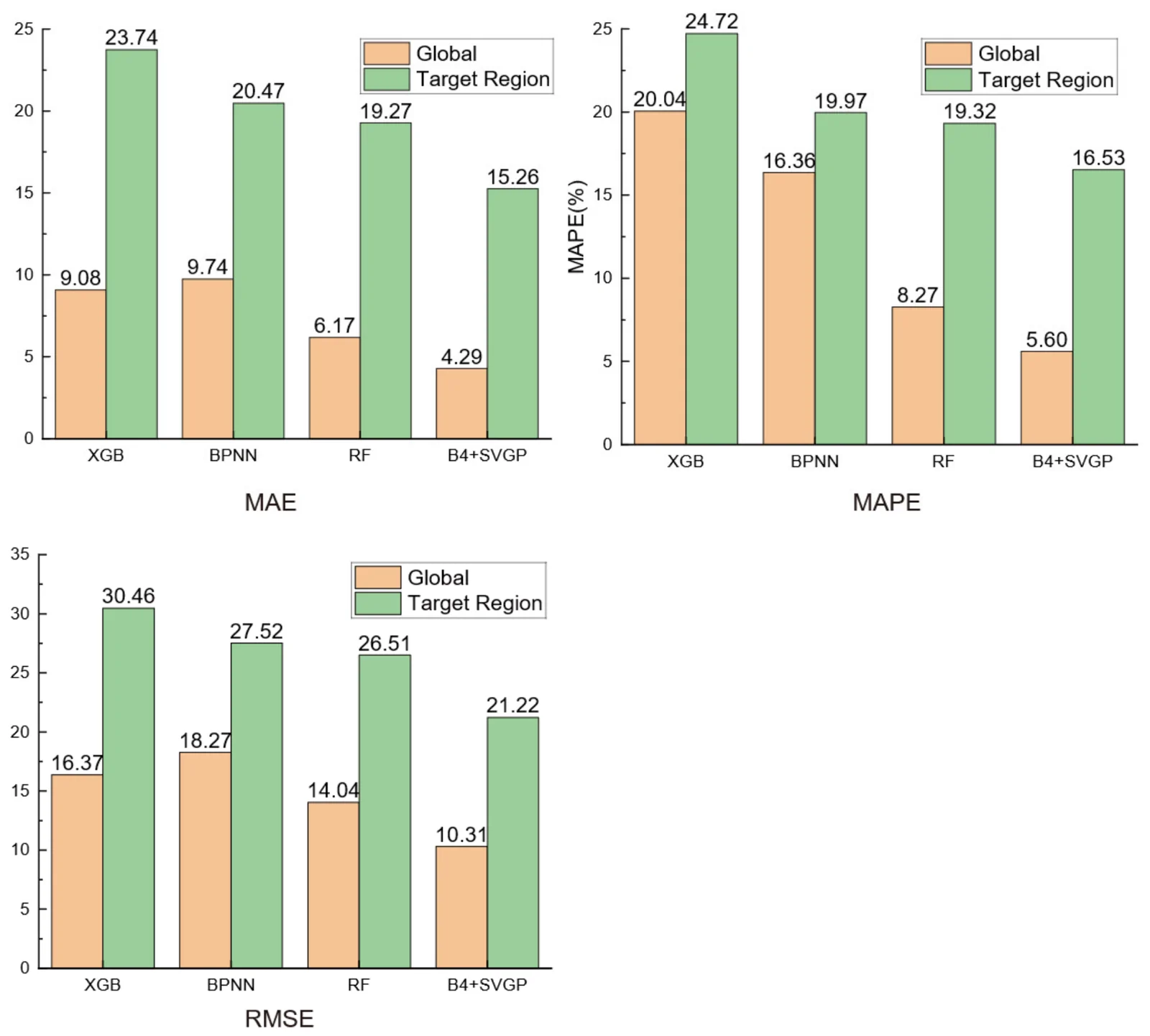
Comparison of evaluation metrics for different models.

To further evaluate the prediction reliability of the models in the target mix proportion region of interest for subsequent optimization (the target region), samples whose decision variables fell within the subsequent multi-objective optimization design domain were selected from the aggregated out-of-fold predictions of the 10-fold cross-validation to form the target-region subset, corresponding to typical low-carbon concrete mix proportions and target service conditions. The MAE, MAPE, and RMSE of each model were then calculated for the global and target regions, and the results are shown in Figure 5. The target-region subset contained 109 samples, accounting for 10.2% of all 1066 samples. This subset was constructed from the aggregated out-of-fold predictions of the 10-fold cross-validation; therefore, the prediction for each sample came from a model that had not been trained on that sample. The errors of all models in the target region were significantly higher than those in the global region, reflecting that the creep compliance of the target-region samples was generally larger and concentrated in a high-value interval with relatively sparse training data, making prediction more challenging. Nevertheless, B4+SVGP retained a relative advantage in the target region, with lower MAE, MAPE, and RMSE than the other three models. Its RMSE increased from 10.31 × 10^−6^/MPa globally to 21.22 × 10^−6^/MPa in the target region. Although this absolute increase was relatively large, the RMSE remained approximately 20% lower than that of the second-best model, RF (26.51 × 10^−6^/MPa), and approximately 23% lower than that of BPNN. These results indicate that, within the same record-level out-of-fold prediction set, B4+SVGP had the lowest error in the target region and exhibited favorable cross-record prediction stability, providing a relatively reliable creep objective for subsequent Pareto optimization.

Based on the above results, the rationale for selecting B4+SVGP as the surrogate model for subsequent optimization is summarized from five perspectives. (1) Prediction accuracy: under record-level validation, B4+SVGP achieved the best average values for all four metrics and retained a relative advantage in the target region, providing a relatively reliable basis for calculating the optimization objective. (2) Cross-record prediction stability: in the sparse target-region scenario, which contained only 109 samples and accounted for approximately 10.2% of the original data, B4+SVGP maintained a 10-fold cross-validation average R^2^ of 0.9660, whereas the errors of the purely data-driven models increased more noticeably on samples not used for training, indicating comparatively limited cross-record prediction stability. (3) Uncertainty diagnosis: SVGP can output predictive variance through variational inference; model comparison and optimization-objective calculation in this study use mean predictions, and predictive variance serves mainly as auxiliary diagnostic information for identifying sparse-data regions and does not enter model selection, constraints, or optimization objectives. The optimization workflow therefore currently relies on mean predictions, and uncertainty-aware optimization can be developed on this basis in future work. (4) Physical consistency: B4 provides the dominant trend and SVGP corrects systematic bias; the physical consistency of the final model must be evaluated through diagnostics rather than enforced through formula-level hard constraints. (5) Small-sample stability: residual learning reduces the modeling target from a highly nonlinear compliance surface to a residual surface with smaller amplitude and smoother variation, significantly reducing dependence on sample size. It should be noted that the XGBoost model in Section 5 is used for creep compliance prediction comparison, whereas the XGBoost strength surrogate used in Section 7 takes mix proportion parameters as inputs and predicts 28-day compressive strength; their purposes, input features, and training targets are different.

## 6. Sensitivity Analysis

After B4+SVGP was selected as the optimization surrogate model, its prediction logic needed to be further interpreted to confirm that the key variables on which it relies are consistent with concrete creep mechanisms. This study uses the SHAP method to decompose the predicted creep compliance of each sample into the sum of the contributions of the input features and uses the mean absolute contribution over all samples as the global feature importance measure. It should be noted that SHAP explains the total prediction of B4+SVGP rather than the residual because the optimization decisions are based on the complete prediction output, including the physical prior.

Shapley values were estimated using an approximate sampling method with the training set as the background dataset to balance computational feasibility and result stability. Because some features are strongly correlated, such as W/B and fc28, and V/S and section dimensions, contributions among collinear features may be absorbed by one another. When strong collinearity is present or the feature set changes, the SHAP feature ranking may also change. Therefore, the feature importance discussed below should be interpreted as a descriptive contribution ranking under the current feature set and model, rather than as a causal effect among variables. The physical discussion below is an interpretation of model-output trends and does not constitute material-level causal proof.

**Figure 6:**
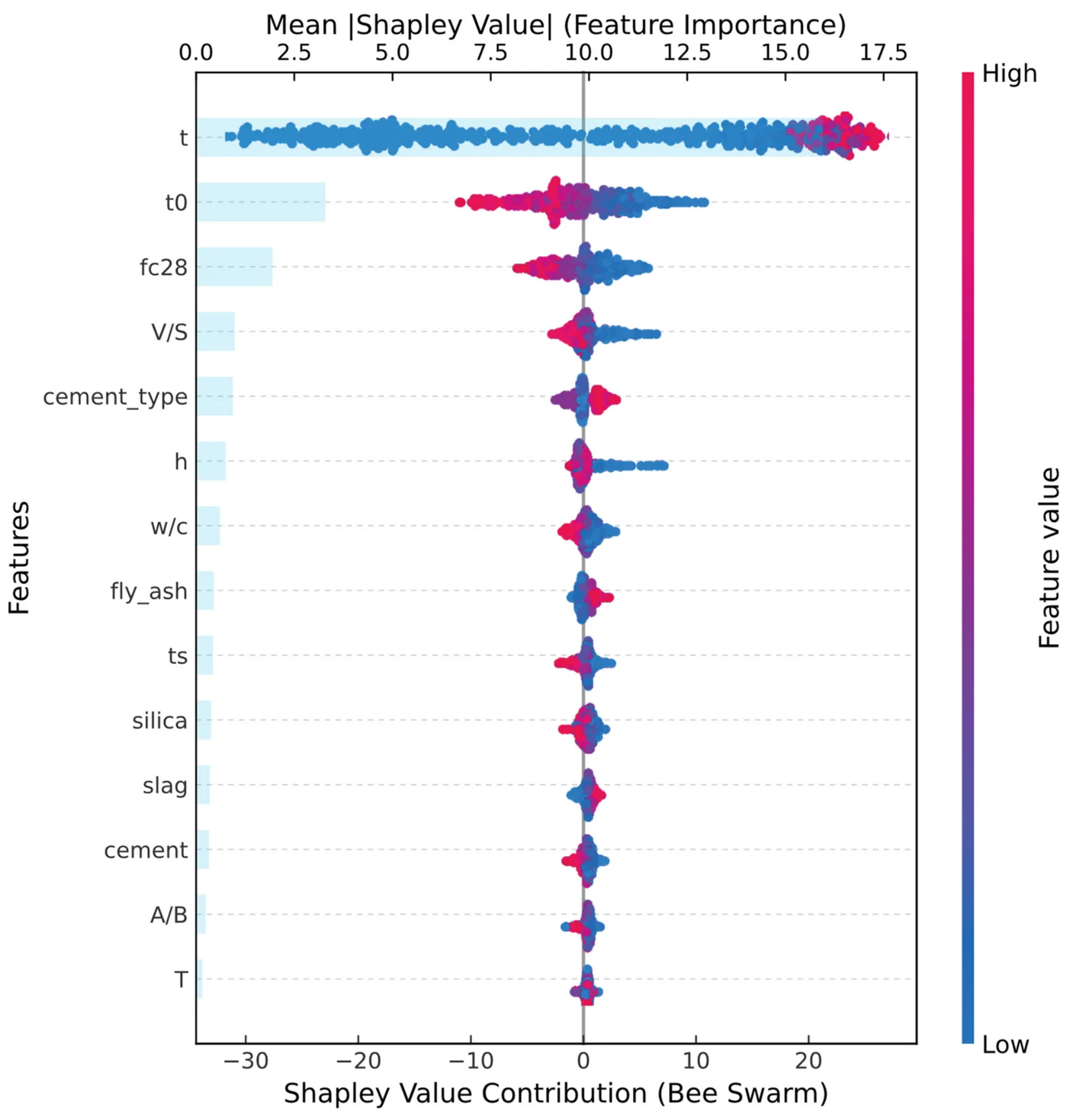
SHAP feature importance and beeswarm contribution plot for the B4+SVGP model.

Figure 6 presents both the global mean Shapley feature importance and the SHAP contribution distributions for each feature. In the upper bar chart, features are ranked by mean absolute contribution. In the lower beeswarm plot, point color represents the feature value (blue for low and red for high), and the horizontal axis represents the SHAP contribution, with positive values driving the predicted creep compliance higher and negative values driving it lower. The upper bar chart shows a pronounced long-tail distribution of feature importance. The mean absolute contribution of loading duration t reached 17.5 × 10^−6^/MPa, approximately 2.3 times that of the second most important feature, age at loading t0 (7.5 × 10^−6^/MPa), while the remaining features were all below 5 × 10^−6^/MPa. These results indicate that time-related variables dominate the model predictions.

The contribution of loading duration t tended toward positive values as t increased. Samples with low t (blue) had SHAP values concentrated in the negative region, whereas samples with high t (red) extended to approximately +25 × 10^−6^/MPa, indicating that a longer loading duration resulted in higher predicted creep compliance. This trend is consistent with the creep mechanisms of adsorbed-water migration and C-S-H interlayer sliding accumulating over time under sustained stress [19,20,21]; t was therefore the most significant positive contributor in the model.

The contribution of age at loading t0 was negative overall and became more negative as t0 increased. Samples with high t0 (red) fell clearly below −25 × 10^−6^/MPa. This trend is consistent with the mechanism that a higher age at loading and a higher degree of hydration densify the microstructure and inhibit the creep rate [19,20,21]; t0 thus predominantly contributed negatively. Considering t and t0 together, the time dimension is the primary factor determining creep compliance.

Among the material parameters, the 28-day compressive strength fc28 had the largest contribution (approximately 4.5 × 10^−6^/MPa). Its high-value samples (red) tended toward negative contributions, whereas low-value samples (blue) tended toward positive contributions, indicating that higher-strength samples generally corresponded to lower predicted creep compliance. This trend is consistent with the general mechanism that a denser matrix pore structure and a smaller creep-susceptible cementitious volume reduce creep compliance [19,20,21], and it also agrees with the model behavior in which strength comprehensively reflects the effects of the water-to-binder ratio and the hydration degree of the cementitious system. It is noteworthy that the independent contribution of W/B was clearly small and had mixed signs. This may be related to its strong correlation with fc28 and the redistribution of contributions among collinear features; therefore, this result cannot be used to infer that the water-to-binder ratio has little effect on creep compliance.

The contributions of the remaining features, including V/S, cement type, and the replacement ratios of supplementary cementitious materials, were concentrated within ±5 × 10^−6^/MPa and distributed symmetrically around zero. Their effects on creep compliance were much smaller than those of loading duration and age at loading, and they therefore did not constitute major drivers in the optimization after the service conditions were fixed.

The above ranking is consistent with creep mechanisms and previous studies. The B4 model, which is based on solidification theory and microprestress relaxation, identifies loading duration, age at loading, and compressive strength as the dominant variables controlling creep evolution. The SHAP-based analysis of the creep behavior of low-carbon concrete by Bouras and Li [6] also identified loading duration as the most significant parameter affecting creep behavior, followed by age at loading. The feature contribution ranking obtained for B4+SVGP in this study is consistent with these findings, providing empirical evidence for the consistency between model behavior and known creep mechanisms. This ranking is also highly consistent with the top five influential parameters identified by Liang et al. [33] using an ensemble machine-learning model and SHAP analysis on the same NU database: loading duration, compressive strength, age at loading, relative humidity, and temperature. The significance of this analysis is not to rediscover these dominant factors, but to quantify their relative contributions in the proposed hybrid model and provide an interpretability check within the data domain.

## 7. Multi-Objective Optimization of Low-Carbon Concrete Mix Proportions Based on B4+SVGP

Reducing carbon emissions while ensuring long-term creep performance represents a key trade-off in low-carbon concrete mix proportion design. To achieve the optimal trade-off between these objectives, this study uses the B4+SVGP surrogate model that performed best under record-level validation as the evaluation kernel for the creep objective and embeds it in NSGA-II. With net carbon emissions and 1000-day creep compliance as the two objectives, the Pareto-optimal set of mix proportions satisfying the constraints is solved within the surrogate-model objective space.

### 7.1. Complete Mapping from Optimization Variables to Surrogate-Model Inputs

Let the NSGA-II candidate solution be

(24) $$\begin{array}{c} x=(x_{w},\;x_{B},\;x_{FA},\;x_{SL},\;x_{SF}) \end{array}$$

where x_w_ is the water-to-binder ratio, x_B_ is the total binder content, and x_FA_, x_SL_, and x_SF_ are the fly ash, slag, and silica fume replacement ratios, respectively. For each candidate solution, the material quantities per unit volume of concrete are first calculated as follows:

(25) $$\begin{array}{c} m_{w}=x_{w}\;x_{B} \end{array}$$

(26) $$\begin{array}{c} m_{FA}=x_{B}\;x_{FA},\;m_{SL}=x_{B}\;x_{SL},\;m_{SF}=x_{B}\;x_{SF} \end{array}$$

(27) $$\begin{array}{c} m_{c}=x_{B}(1-x_{FA}-x_{SL}-x_{SF}) \end{array}$$

where m_w_, m_c_, m_FA_, m_SL_, and m_SF_ are the mixing water, cement, fly ash, slag, and silica fume contents, respectively, all in kg/m^3^.

The aggregate-to-binder ratio is defined as the ratio of total aggregate mass to total binder mass. Fine and coarse aggregates are not distinguished, and air content is not introduced; the aggregate is treated as a total mass. ma is determined by the absolute volume balance of concrete:

(28) $$\begin{array}{c} 1=\frac{m_{w}}{\rho_{w}}+\frac{m_{c}}{\rho_{c}}+\frac{m_{FA}}{\rho_{FA}}+\frac{m_{SL}}{\rho_{SL}}+\frac{m_{SF}}{\rho_{SF}}+\frac{m_{a}}{\rho_{a}} \end{array}$$

where $\rho_{w}$, $\rho_{c}$, $\rho_{FA}$, $\rho_{SL}$, $\rho_{SF}$ and $\rho_{a}$ are the densities of mixing water, cement, fly ash, slag, silica fume, and total aggregate, respectively. The density parameters follow the unified conversion parameters used in the feature-construction stage of the experimental database to ensure consistent definitions between model training and optimization.

For each candidate solution, the above relationships provide all inputs required by the B4+SVGP creep surrogate model. The way in which each input is obtained during optimization is summarized in Table 6.

The 28-day compressive strength was predicted by a separately trained XGBoost strength surrogate model. This model uses seven mixture-related inputs: water-to-binder ratio, aggregate-to-binder ratio, cement content, cement type, silica fume content, fly ash content, and slag content, with 28-day compressive strength as the output. The dataset contains 532 records, of which 372 were used for training and 160 for testing, giving a training/test ratio of approximately 7:3; the data were split by unique mix proportion to prevent the same mix proportion from appearing in both subsets. On the test set, the model achieved R^2^ = 0.9765, RMSE = 3.69 MPa, MAE = 1.21 MPa, and MAPE = 2.58%. The XGBoost strength surrogate was adopted because fixed-form empirical strength equations show relatively large scatter in multi-component SCM low-carbon concrete systems, whereas XGBoost can capture nonlinear interactions among the water-to-binder ratio, cementitious composition, and aggregate content. After independent testing, the model was used for strength estimation and constraint evaluation during optimization. The data-split and training random seeds for this XGBoost strength surrogate were 54 and 55, respectively. During optimization, the candidate mix proportions have no measured 28-day compressive strength, and fc,28 used for both constraint evaluation and the B4+SVGP creep objective is provided by this XGBoost model. Its prediction error may therefore enter both feasibility classification and creep prediction, so candidates close to the 30 or 60 MPa constraint bounds are interpreted with an appropriate error margin. Given the relatively low test error and the restriction of the optimization domain to the training-data envelope, the resulting Pareto front serves as a relative optimization result under the current two-stage surrogate framework for candidate screening, with experimental confirmation to follow before engineering application. The main settings are listed in Table 7.

### 7.2. Optimization Model, Objectives, and Constraints

The decision variables are five mix proportion design parameters: water-to-binder ratio, total binder content, fly ash replacement ratio, slag replacement ratio, and silica fume replacement ratio. To unify the optimization environment, the decision variables were restricted to the empirical envelope corresponding to the training data to reduce the risk of surrogate-model extrapolation. The environmental and geometric parameters were fixed as follows: a 100 mm × 100 mm × 400 mm square prismatic specimen (corresponding to a volume-to-surface-area ratio of 22.22), normal-setting Portland cement (R), drying age and age at loading of 28 days, relative humidity of 65%, and ambient temperature of 21.5 °C. This fixed-parameter setup is consistent with the sample-size attenuation scenario in Section 2.4, ensuring that the optimization domain lies largely within the empirical envelope of the training data. During optimization, the creep objective is given by the mean prediction of B4+SVGP; because the decision variables have been restricted to the training-data coverage domain confirmed by the target-region analysis in Section 5, the Pareto search is mainly located within this domain to reduce extrapolation risk. This domain defines a consistent computational boundary for candidate screening, and subsequent experiments can further confirm engineering performance on this basis.

The first objective is 1000-day net carbon emissions, obtained by subtracting the carbonation sink during service from the production-stage emissions in Section 4. The second objective is 1000-day creep compliance, predicted directly by the B4+SVGP surrogate model. Both objectives use 1000 days after loading as the unified assessment time, making carbon emissions and creep performance comparable in the time dimension.

The optimization must satisfy physical feasibility and strength requirements: the total binder content must be 300–550 kg/m^3^; the water-to-binder ratio must be 0.30–0.60; the combined mass of fly ash, slag, and silica fume as a proportion of total binder must be less than 50%; the individual replacement ratios of fly ash, slag, and silica fume must be less than 35%, 50%, and 15%, respectively; and the 28-day compressive strength predicted by the XGBoost strength surrogate must be between 30 and 60 MPa. Candidate solutions that violate the mix-proportion ranges or strength constraints are treated as infeasible individuals in NSGA-II, and a penalty function reduces their selection probability. Except for the strength prediction model, all mix-proportion constraints are deterministic bounds and do not involve additional empirical equations.

NSGA-II was used to solve the above bi-objective problem. Its non-dominated sorting and crowding-distance mechanisms maintain solution diversity while ensuring convergence. The creep objective was calculated by the B4+SVGP surrogate model, and the carbon emission objective was calculated directly using the equations above. The main algorithm parameters are listed in Table 8. The seeds for the 10 independent NSGA-II runs were deterministically derived from a master seed s0 = 33 SeedSequence(33).spawn(10) in NumPy 2.2.6; the i-th run uses the first uint32 from generate_state(4) as integer seed si and constructs np.random.default_rng(si), thereby uniquely determining the 10 run seeds.

To assess convergence, this study uses the hypervolume (HV) indicator. Its reference point was set to a value slightly inferior to all non-dominated solutions in the objective space, so that HV increases as the front approaches the ideal point.

**Figure 7:**
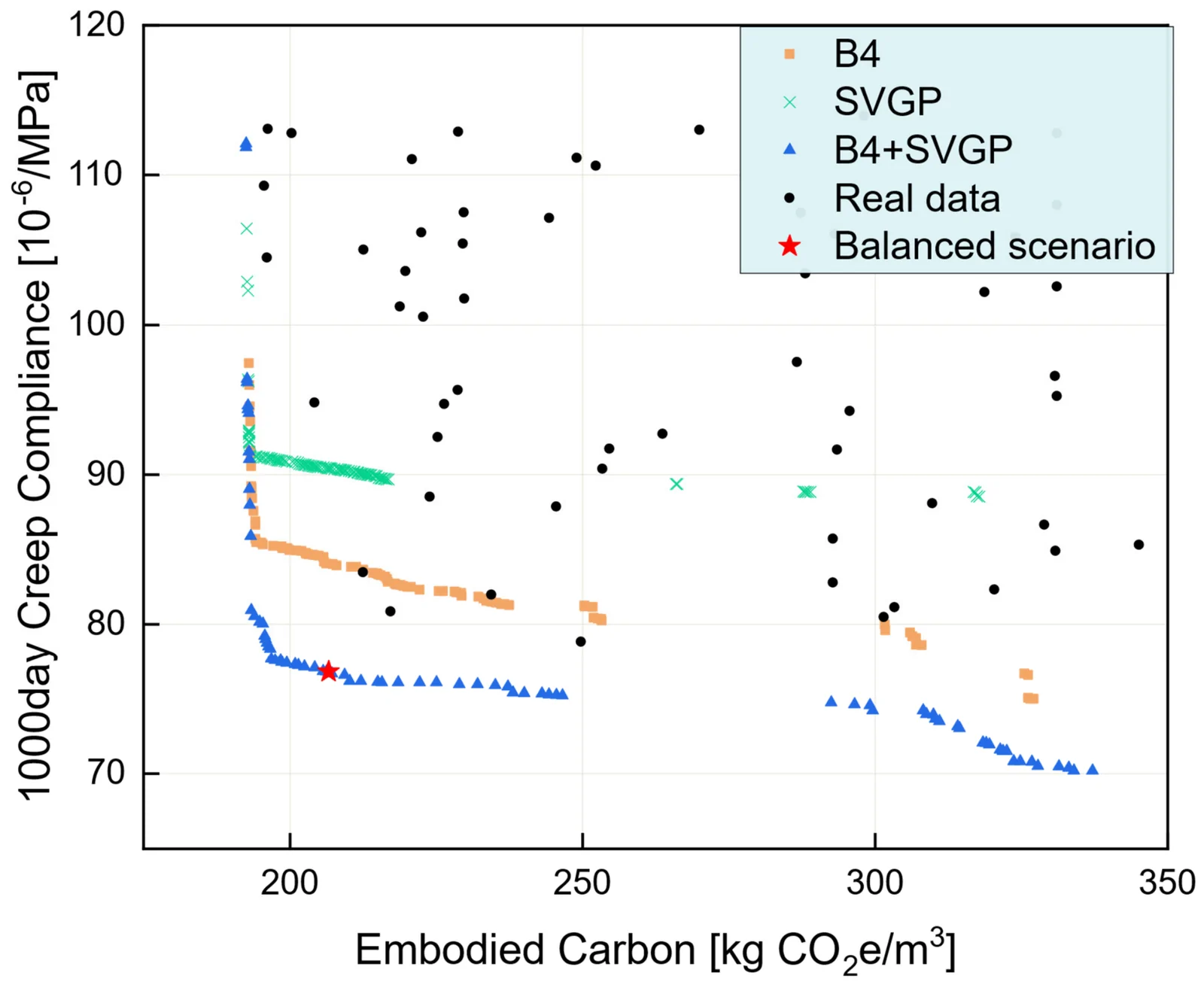
Pareto fronts obtained using different surrogate models.

Figure 7 shows the Pareto fronts obtained after embedding the three surrogate models in NSGA-II. The horizontal axis represents net carbon emissions (kg CO_2_e/m^3^), and the vertical axis represents 1000-day creep compliance (10^−6^/MPa). The differences in the shape and position of the three fronts reflect the influence of the surrogate model on the optimization results. To provide an external reference for the front results, held-out experimental data are further overlaid in the figure; their creep compliance values are the measured values at 1000 days or values interpolated from measured creep curves under matched conditions. Most of the held-out experimental points lie above or adjacent to the B4+SVGP front, and the front approximately forms their lower envelope, indicating that the optimized front is generally consistent with the held-out experimental records and can serve as a reference for interpreting the front position.

The B4+SVGP front exhibits a pronounced L shape. When net carbon emissions increase from approximately 192 kg/m^3^ to approximately 205 kg/m^3^, creep compliance decreases rapidly from approximately 97 × 10^−6^/MPa to approximately 76 × 10^−6^/MPa. After net carbon emissions exceed approximately 205 kg/m^3^, creep compliance decreases only slowly from approximately 76 × 10^−6^/MPa to approximately 70 × 10^−6^/MPa, and the reduction becomes substantially smaller. This indicates a clear inflection point at approximately 205 kg/m^3^, beyond which further increases in carbon emissions provide very limited improvement in creep compliance.

In contrast, the B4 front shifts upward overall, and its creep compliance is higher than that of B4+SVGP at the same carbon emissions. The SVGP front is nearly horizontal, with creep compliance remaining stable at approximately 91 × 10^−6^/MPa; therefore, a reduction in creep compliance can hardly be achieved by increasing carbon emissions.

The differences among the three models originate from their extrapolation capability in the sparse target region. The B4 model was calibrated using ordinary Portland cement data and systematically overestimates creep compliance for systems with high contents of fly ash, slag, and silica fume; consequently, its front is generally higher. Residual learning corrects this bias and gradually lowers the creep predictions, causing the B4+SVGP front to lie below the B4 front. Pure SVGP lacks physical prior constraints and deteriorates during extrapolation in the target region, where samples account for only 10.2% of the full dataset and are concentrated in a sparse high-value interval; it consequently outputs nearly constant high creep compliance, flattening the front. The ranking is consistent with the target-region error results in Section 5, indicating that the differences among surrogate models are manifested consistently in cross-validation and in the optimization space. This consistency primarily supports the interpretation of prediction behavior within the current surrogate-model framework and does not directly establish the superiority or inferiority of material physical performance.

**Figure 8:**
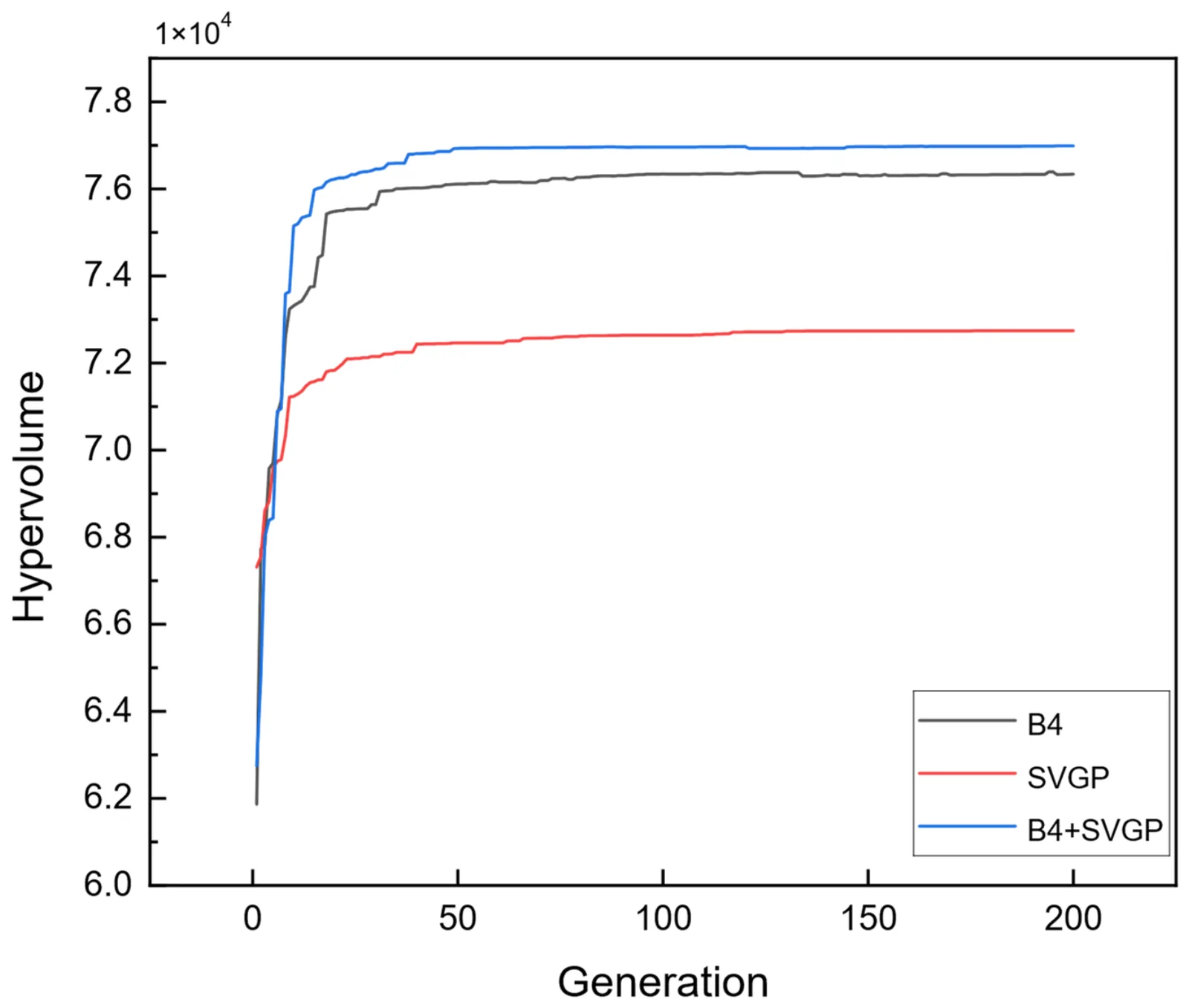
Hypervolume convergence curves of different surrogate models.

Figure 8 shows the convergence of hypervolume with the number of generations after embedding the three surrogate models in NSGA-II. All curves increase rapidly in the early stage and then level off, indicating that the algorithms converge stably to a non-dominated front. B4+SVGP had the highest final hypervolume, followed by B4, while SVGP had the lowest value, consistent with the front dominance relationships in Figure 7. These results indicate that, under the current surrogate models and optimization settings, the non-dominated solution set obtained by B4+SVGP has a higher hypervolume and more complete objective-space coverage. This result mainly reflects the computational front quality generated by the surrogate model, and its material-level significance still requires experimental verification.

Table 9 presents TOPSIS decision-support scenarios under different weight priorities. The carbon emissions/creep weight combinations are 0.7/0.3 (carbon-emission-oriented), 0.5/0.5 (balanced), and 0.3/0.7 (creep-oriented), covering the typical engineering preference range from environment-driven to performance-driven requirements. TOPSIS uses vector normalization to eliminate dimensional differences between the two objectives and ranks solutions by their relative closeness to the positive and negative ideal solutions. As the weight preference shifts from carbon-emission-oriented to balanced and then to creep-oriented, net carbon emissions increase from 194.77 to 206.68 and 254.32 kg/m^3^, while creep compliance decreases from 80.16 to 76.78 and 75.21 × 10^−6^/MPa, showing a clear monotonic trade-off; all three solutions lie on the B4+SVGP front.

The balanced-weight solution (5:5) is located at the front inflection point, that is, the red star in Figure 7. Compared with the creep-oriented solution (3:7), it reduces net carbon emissions by approximately 18.7% at the cost of only approximately 2.1% higher creep compliance. Compared with the carbon-emission-oriented solution (7:3), it increases net carbon emissions by approximately 6.1% while reducing creep compliance by approximately 4.2%. Therefore, the balanced-weight scenario provides a favorable trade-off between carbon emissions and creep and corresponds to a water-to-binder ratio of 0.4227, total binder content of 300.03 kg/m^3^, fly ash replacement ratio of 6.76%, slag replacement ratio of 6.70%, and silica fume replacement ratio of 14.82%. This solution satisfies all constraints and lies within the training-data coverage. The balanced-weight scenario (5:5) represents one engineering compromise under equal weighting; the final choice in practical engineering should also consider project objectives, material availability, cost, construction conditions, and applicable standards. The three weighting scenarios correspond to different engineering requirements and can provide references for mix proportion decisions; in practice, a suitable mix proportion can be selected from the complete Pareto set according to project-specific preferences. This trade-off trend is consistent with previous conclusions on the effect of supplementary cementitious material contents on creep performance, indicating that the optimized mix proportions are consistent with material mechanisms. Before these mixtures can be recommended for practical engineering applications, experimental verification of fresh-state properties (e.g., workability and setting time), compressive strength, and long-term creep behavior should be conducted using the actual raw materials. Only after the results meet the relevant design and code requirements should they be considered engineering recommendations. The surrogate-model results can support screening and trade-off analysis but do not replace final performance assessment.

## 8. Conclusions

To meet the need for coordinated design of the environmental performance and long-term mechanical performance of low-carbon concrete, this study proposed a concrete creep prediction method based on a physical prior and residual learning and embedded it in a multi-objective optimization framework. An intelligent design system for low-carbon concrete mix proportions was established with net carbon emissions and creep performance as the two objectives. The main findings are as follows:

(1) To address the scarcity of creep test data for low-carbon concrete and the insufficient generalization stability and physical consistency of purely data-driven models under small-sample conditions, a hybrid modeling strategy was proposed in which the theoretical values of the B4 creep model serve as the physical prior backbone and Gaussian process regression learns the residual. Global linear calibration was used to establish residual labels, and a sparse variational Gaussian process (SVGP) with an ARD Matérn-5/2 kernel was adopted. A lower-bound constraint on the length scale and a Gamma prior effectively suppressed kernel collapse, achieving physical-prior-guided creep prediction in small-sample, high-dimensional sparse scenarios.
(2) Under a unified dataset, feature set, and training protocol, B4+SVGP had the lowest average error metrics under the unified record-level 10-fold cross-validation: R^2^ reached 0.9660, RMSE was 10.305 × 10^−6^/MPa, MAE was 4.291 × 10^−6^/MPa, and MAPE was 5.60%, outperforming random forest (R^2^ = 0.891), XGBoost, and BPNN (R^2^ = 0.816). In the target mix proportion region of interest for optimization, its RMSE was approximately 20% lower than that of the second-best model, further increasing its relative advantage. These results mainly support the relative predictive advantage under the current database and record-level protocol, while applicability at the independent mixture or experimental-series level can be further examined in future work.
(3) SHAP interpretability analysis showed that loading duration was the most significant feature affecting creep compliance, followed by age at loading and 28-day compressive strength. The feature contribution ranking was consistent with the physical mechanisms of the B4 model based on solidification theory and microprestress relaxation as well as with previous studies, providing empirical evidence for the consistency between model behavior and known creep mechanisms; the time dimension is the primary factor governing the evolution of creep compliance. The significance of this result is to provide a quantitative interpretability check for the hybrid model, rather than to claim the discovery of previously unknown factors.
(4) The B4+SVGP model that performed best under record-level validation was embedded in NSGA-II as the creep objective kernel, and low-carbon concrete mix proportions were solved with net carbon emissions and 1000-day creep compliance as the two objectives. The Pareto front exhibited a pronounced L shape with an inflection point at net carbon emissions of approximately 205 kg/m^3^. Before this point, increasing carbon emissions rapidly reduced creep compliance from approximately 97 × 10^−6^/MPa to approximately 76 × 10^−6^/MPa; after this point, the marginal improvements diminished significantly. TOPSIS provided decision-support scenarios under three weight preferences, and the balanced scenario (5:5) corresponded to a water-to-binder ratio of 0.4227, total binder content of 300.03 kg/m^3^, fly ash replacement ratio of 6.76%, slag replacement ratio of 6.70%, and silica fume replacement ratio of 14.82%, located at the front inflection point and achieving a coordinated trade-off between environmental and long-term performance in the surrogate-model-predicted objective space. In the optimization stage, the 28-day compressive strength was predicted by the XGBoost strength surrogate validated on an independent test set and, together with the mix proportion parameters, formed the complete B4+SVGP input, ensuring reproducibility of the optimization workflow.
(5) The proposed method and optimization results mainly apply to the database-covered parameter range; given the simplified carbonation model and incomplete representation of admixtures, the conclusions should be examined under wider conditions. The folds are mutually exclusive at the record level, but correlated records from the same mixture or experimental series may still appear in different folds. The reported accuracy is therefore interpreted within the record-level validation scope, and extrapolation to independently sampled mixtures should be treated with caution; the magnitude of this effect cannot be quantified from the available metadata. Because all models use the same folds and all preprocessing and tuning are confined to training folds, the comparison remains internally consistent within this scope. Grouped validation by mix proportion or literature source should be prioritized once reliable identifiers or independent data become available. TOPSIS selections should reflect project-specific weights and conditions, and the optimized mixtures remain computational candidates. Their fresh-state properties, compressive strength, and long-term creep behavior should be experimentally verified using actual raw materials, and recommendation for engineering applications should follow only after the results satisfy relevant design and code requirements. Predictive uncertainty and expanded data can further strengthen applicability.

## Figures and Tables

**Table 1:** Statistical characteristics and units of input parameters.

	Unit	Min	Max	Mean	SD
W/B	Unitless	0.2	0.74	0.36	0.12
A/B	Unitless	1.22	10.8	3.90	1.53
c	kg/m^3^	183.8	570	425.8	54.7
silica	Unitless	0	0.18	0.053	0.053
fly ash	Unitless	0	0.47	0.01	0.014
slag	Unitless	0	0.39	0.02	0.023
t_L	d	1	365	55.2	160.4
t_D	d	1	28	25.7	6.69
RH	%	20	75	51.6	9.36
V/S	mm	10.6	46.6	22.9	9.20
T	°C	18	23	20.2	2.45
fc28	MPa	30.3	118.9	74.9	24.97
t	d	0	6768	440.9	731.3

Note: silica fume, fly ash, and slag replacement ratios are expressed as mass fractions (dimensionless); 0.18 denotes 18% by mass. Relative humidity RH is expressed as a percentage; 20, 75, and 51.6 denote 20%, 75%, and 51.6%, respectively.

**Table 2:** Variance inflation factors (VIFs) of input features.

Features	VIF	Features	VIF
W/B	6.46	t_D	1.20
fc28	4.74	t_L	1.10
c	4.46	silica	1.85
A/B	3.96	slag	2.08
fly ash	3.39	V/S	2.07
RH	2.48	t	1.34
T	1.52		

**Table 3:** One-hot encoding of cement type.

Cement Type	R	RS	SL
R	1	0	0
RS	0	1	0
SL	0	0	1

**Table 4:** Carbon emission factors of raw materials.

Raw Material	Carbon Emission Factor (kg CO_2_e/kg)
Ordinary Portland cement	0.80
Fly ash	0.030
Ground granulated blast-furnace slag	0.07
Silica fume	0.35
Aggregate	0.003
Mixing water	0.00018

**Table 5:** Hyperparameters and training settings of each model.

Model	Architecture	Optimizer	Batch Size	Number of Epochs	Early-Stopping Rule
BPNN	16-128-64-32-1 MLP; ReLU hidden layers	Adam (lr = 5 × 10^−4^)	32	148	Not used
RF	200 trees; max_depth = 10; min_samples_split = 2	—	—	—	—
XGBoost	100 trees; max_depth = 5; learning_rate = 0.10; subsample = 0.80	—	—	—	—
SVGP	200 inducing points; Matérn-5/2 ARD kernel; linear mean function	Adam (lr = 0.01)	Full batch	300	ELBO-based early stopping; patience = 40

**Table 6:** Complete mapping from optimization variables to B4+SVGP model inputs.

B4+SVGP Input	Obtained in the Optimization Stage
Water-to-binder ratio W/B	Decision variable x_w_
Aggregate-to-binder ratio A/B	Calculated from material quantities and absolute volume balance
Cement content c	x_B_(1—x_FA_—x_SL_—x_SF_)
Cement type	Fixed as type R
Silica fume replacement ratio	Decision variable x_SF_
Fly ash replacement ratio	Decision variable x_FA_
Slag replacement ratio	Decision variable x_SL_
Age at loading	Fixed at 28 days
Drying age	Fixed at 28 days
Relative humidity	Fixed at 65%
Volume-to-surface-area ratio V/S	Fixed at 22.22
Ambient temperature	Fixed at 21.5 °C
28-day compressive strength fc28	Predicted by the XGBoost strength surrogate
Loading duration t	Fixed at 1000 days

**Table 7:** Data split, performance metrics, and main hyperparameters of the XGBoost strength surrogate model.

Item	Setting/Result
Training/test split	7:3, split by unique mix proportion
R^2^	0.9765
RMSE	3.69 MPa
MAE	1.21 MPa
MAPE	2.58%
max_depth	3
n_estimators	300
learning_rate	0.15
subsample	1.0
colsample_bytree	0.8
min_child_weight	3

**Table 8:** Parameter settings of the NSGA-II algorithm.

Parameter	Value
Population size	100
Maximum number of generations	200
Crossover probability	0.90
Mutation probability	0.20
Crossover distribution index	20
Mutation distribution index	20
Number of inducing points	200
Number of independent runs	10

**Table 9:** TOPSIS decision-support scenarios under different weight preferences.

Weight Preference	w/b	Total Binder Content (kg/m^3^)	Fly Ash Replacement Ratio	Slag Replacement Ratio	Silica Fume Replacement Ratio	Net Carbon Emissions (kg CO_2_e/m^3^)	1000-Day Creep Compliance (10^−6^/MPa)
Carbon-emission-oriented	0.3936	300.68	0.0615	0.1240	0.1473	194.77	80.16
Balanced	0.4227	300.03	0.0676	0.0670	0.1482	206.68	76.78
Creep-oriented	0.4970	321.16	0.0087	0.0004	0.1484	254.32	75.21

Note: The weights are given as (carbon emissions:creep); the three scenarios are 7:3, 5:5, and 3:7, respectively.

## Data Availability

The original contributions presented in this study are included in the article. Further inquiries can be directed to the corresponding author.
